# Modeling the Circadian Control of the Cell Cycle and Its Consequences for Cancer Chronotherapy

**DOI:** 10.3390/biology12040612

**Published:** 2023-04-18

**Authors:** Courtney Leung, Claude Gérard, Didier Gonze

**Affiliations:** Unité de Chronobiologie Théorique, Faculté des Sciences CP 231, Université Libre de Bruxelles, Bvd du Triomphe, 1050 Bruxelles, Belgium

**Keywords:** cell cycle, circadian entrainment, heterogeneity, cancer chronotherapy

## Abstract

**Simple Summary:**

The circadian clock controls many physiological processes including the cell division cycle. Healthy cells thus have a higher propensity to divide at certain times during the day. In many cancer cells, the circadian entrainment of the cell division cycle is impaired or lost, due to a disrupted clockwork. Here, we use a computational model describing the molecular network governing the progression into the successive phases of the cell cycle and investigate, through numerical simulations, the consequences of the circadian control on the dynamics of the cell cycle. Our results allow us to predict the optimal timing for the application of anti-cancer drugs that target specific phases of the cell division cycle and highlight the importance of better characterization of cellular heterogeneity and synchronization in cell populations in order to design successful chronopharmacological protocols.

**Abstract:**

The mammalian cell cycle is governed by a network of cyclin/Cdk complexes which signal the progression into the successive phases of the cell division cycle. Once coupled to the circadian clock, this network produces oscillations with a 24 h period such that the progression into each phase of the cell cycle is synchronized to the day–night cycle. Here, we use a computational model for the circadian clock control of the cell cycle to investigate the entrainment in a population of cells characterized by some variability in the kinetic parameters. Our numerical simulations showed that successful entrainment and synchronization are only possible with a sufficient circadian amplitude and an autonomous period close to 24 h. Cellular heterogeneity, however, introduces some variability in the entrainment phase of the cells. Many cancer cells have a disrupted clock or compromised clock control. In these conditions, the cell cycle runs independently of the circadian clock, leading to a lack of synchronization of cancer cells. When the coupling is weak, entrainment is largely impacted, but cells maintain a tendency to divide at specific times of day. These differential entrainment features between healthy and cancer cells can be exploited to optimize the timing of anti-cancer drug administration in order to minimize their toxicity and to maximize their efficacy. We then used our model to simulate such chronotherapeutic treatments and to predict the optimal timing for anti-cancer drugs targeting specific phases of the cell cycle. Although qualitative, the model highlights the need to better characterize cellular heterogeneity and synchronization in cell populations as well as their consequences for circadian entrainment in order to design successful chronopharmacological protocols.

## 1. Introduction

Every day, several tens of billion cells die and are replaced by new cells in human adults [1]. Tissue homeostasis is maintained throughout the lifespan by a tight control of the balance between cell death, proliferation, and differentiation. The mammalian cell division cycle is made up of four phases: DNA replication (S phase) and mitosis (M phases) are separated by gap phases (G1 and G2 phases) during which RNAs and proteins are synthesized. The progression along the successive phases of the cell cycle is dictated by a network of enzymes known as cyclin-dependent kinases (Cdks), reversibly activated through phosphorylation/dephosphorylation and through binding to specific cyclins [2]. This network is tightly controlled by numerous factors, including growth factors, molecular checkpoints, and the circadian clock. Dysregulation of this control system, caused by mutations in the components of the signaling pathways for example, may lead to uncontrolled cell division and to cancer. The duration of the cell division cycle is characterized by stochastic variability, but the coupling of the Cdk network to the circadian clock enables cells in some tissues to divide in a synchronized way, on a 24 h time scale. Disruption of this circadian control in cancer cells leads to less synchronized cell division, a feature exploited to optimize the timing of drug administration [3,4]. Here, we illustrate, through computational modeling, how cell–cell variability and (lack of) circadian entrainment impact chronotherapeutic strategies.

The circadian clock is a timing system which couples physiological processes to daily changes in the external environment [5,6]. Circadian rhythms are autonomously generated in the suprachiasmatic nucleus (SCN) of the hypothalamus, but can be entrained in response to external cues, and in particular to the 24 h light–dark cycle. Circadian oscillations are transmitted to peripheral body cells, so that the clock and the clock-controlled physiological processes in many tissues acquire a period of 24 h. At the molecular level, circadian oscillations originate from a network of interlocked gene regulatory feedback loops. The dominant loop involves the CLOCK/BMAL1 dimer, which activates the transcription of *Per* and *Cry* clock genes, coding for repressors of *Bmal1* and *Clock* transcription [6]. Degradation of PER and CRY proteins resets the clock. Additional loops involving RevErbα and ROR contribute to the generation and robustness of the circadian oscillations [6].

Multiple molecular links between the circadian clock and the cell cycle have been identified. Several genes involved in regulating the formation of cyclin/Cdk complexes are regulated at the transcriptional level by clock components. Matsuo et al., (2003) found that the transcription of *Wee1*, a gene that prevents entry into the M phase of the cell cycle by inhibiting cyclin B/Cdk1 formation, is induced by the CLOCK/BMAL1 dimer [7]. The latter also regulates the transcription of the Cdk inhibitor p21 [8] and represses the oncogene c-Myc that induces the expression of cyclin E [9]. Such coupling to the circadian clock may lead to the entrainment of the cell cycle, which can explain why cell division often operates on a 24 h time scale [10,11].

It was proposed that the circadian clock is a tumor suppressor [12]. Many studies have supported that disruption of the circadian clock, for example through mutations in the clock gene *Per*, increases the risk of tumorigenesis [13]. Shift work and chronic jet lag are risk factors for breast cancer [14]. In cancer cells, the circadian clock is often impaired [15,16,17], so that circadian regulation of the cell cycle is less effective. In some cancer cells the cell cycle may even be decoupled from circadian rhythms [18].

Chronotherapy refers to treatment administration taking into account biological rhythms, in order to optimize effectiveness and minimize toxicity [3,19,20]. Chronotherapy is a promising route to improve anti-cancer treatments as cancer cells often have cell cycles unsynchronized with those of entrained healthy cells. We may thus restrict drug administration to the time windows during which healthy cells are not sensitive to the drug (minimizing toxicity), and hoping that during this period of time a certain proportion of the (desynchronized) cancer cells will be sensitive to the drug (maximizing effectiveness). Paclitaxel and seliciclib have great potential for chronotherapy as they act at particular cell cycle phases. Paclitaxel blocks the depolymerization of microtubules, which results in a Cyclin B/Cdk1 complex that remains active. The targeted cells are blocked in M phase and undergo death through apoptosis [21,22]. Seliciclib inhibits Cdk2, and thus targets cells in G1-S phase [23]. Another good candidate drug is 5-fluorouracil (5-FU), a pyrimidine analog, which acts during DNA replication (S phase), by inhibiting thymidylate synthase and thus interfering with DNA synthesis.

The duration of the cell cycle (and of its respective phases) is variable. Both deterministic and stochastic sources of variability are responsible for this cellular heterogeneity [24]. In particular, stochastic noise in gene expression [25,26,27] and unequal partitioning of cellular components at cell division [28] affect kinetic rates and thereby the dynamics of the cell cycle. Analysis of correlations of single cell division times across lineages also highlights the existence of underlying deterministic factors in generating cell-to-cell variability [29,30]. On the other hand, the coupling and the resulting entrainment of the Cdk network by the circadian clock may reduce cell-to-cell variability and enable cells to divide in synchrony [24]. In cancer cells, it is expected that, due to circadian disruption and variability in the kinetic parameters, cell divisions lose their synchrony.

Computational modeling is a convenient tool to explore the consequences of the coupling between the cell cycle and the circadian clock and to test therapeutic strategies. Gérard and colleagues have devised several computational models based on ordinary differential equations (ODEs) to describe the sequential activation and inactivation of cyclin/Cdk complexes in the network through reversible phosphorylation and synthesis/degradation of cyclins [31,32,33]. These models show that the cell cycle is initiated by an above-threshold level of growth factors. Beyond initiation, the cell cycle network is capable of self-sustained oscillations, corresponding to cell proliferation. This type of models can be used to describe the dynamics at the single-cell level in response to changes in some kinetic parameters (e.g., in response to the application of some drug), to clarify the role of positive regulatory loops in the robustness of the oscillations [33], to identify the conditions of entrainment when coupled to the circadian clock [34,35,36], or to assess the effect of stochastic noise in a cell population [37]. Molecular models, calibrated through fitting to experimental time profiles of concentrations, can be used to predict potential drug targets or to design chronopharmacological protocols [38,39,40,41,42]. Another class of models, based on automata and probabilistic transitions of the cells into the successive phases of the cell cycle, can be used to account for the dynamics of large and heterogeneous cell populations and to simulate the effect of drugs at the population level [43,44,45].

Here, we opted for an ODE-based approach, but, to account for cell–cell heterogeneity, we consider a population of cell cycle oscillators. We used the model proposed by Gérard et al. (2012) [33], to which we incorporated a circadian control on the cell cycle to study the entrainment and synchronization of the cell cycle. We first investigated the properties of the cell cycle in presence of inter-cellular variability. We then determined the conditions for which the cell cycle could be properly entrained to a 24 h cycle as a function of the amplitude of the circadian input and the autonomous cycle period. Next, we simulated the dynamics of the cell cycle in cancer cells in the absence of circadian control and with a low amplitude circadian rhythm. Finally, we investigated the effectiveness and toxicity of anti-cancer drugs (such as paclitaxel or seliciclib) on the cell cycle as a function of administration time. We simulated administrations of anti-cancer drugs at different frequencies to assess the long-term effect of chronomodulated treatments.

## 2. Model

**Figure 1:**
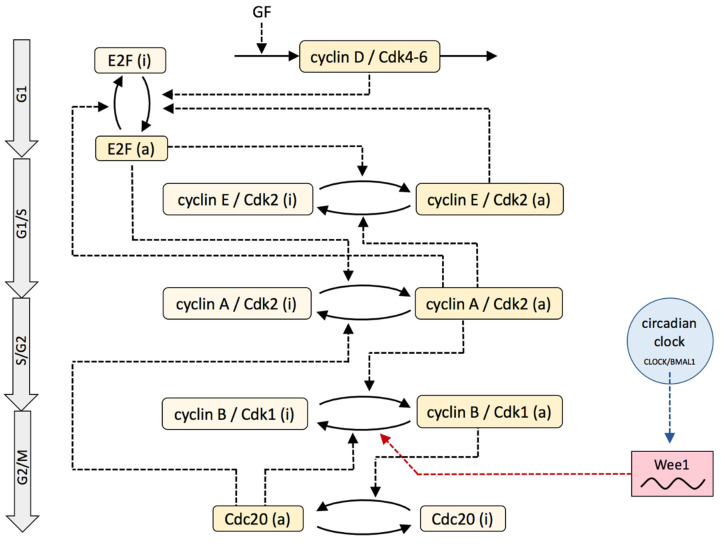
Scheme of the cell cycle model. The model describes the dynamics of the four main cyclin/Cdk complexes, the transcription factor E2F, and the protein Cdc20 [33]. Solid arrows denote synthesis/degradation of the cyclin D/Cdk4-6 complex and activation/deactivation of the other complexes. Dashed arrows indicate the regulations. The dynamics of *Wee1*, whose the synthesis is controlled by the circadian clock, is described by a 24 h period sine function. *Wee1* induces the deactivation of the cyclin B/Cdk1 complex.

The model used in the present study is schematized in Figure 1 [33]. The model is centered on the four main cyclin/Cdk complexes, the transcription factor E2F, and the protein Cdc20. The presence of a growth factor (GF) ensures the synthesis of the cyclin D/Cdk4–6 complex, which promotes progression in the G1 phase. This complex activates the transcription factor E2F, which brings about the synthesis of cyclins E and A, and thereby the activation of the cyclin E/Cdk2 complex at the G1/S transition, and of the cyclin A/Cdk2 complex during the S phase. Cyclin E/Cdk2 also activate E2F, which reinforces the activation by cyclin D/Cdk4–6 and promotes progression to the G1/S transition. Cyclin A/Cdk2 allows progression in S phase and elicits the S/G2 transition by inducing the inactivation of E2F. During G2, cyclin A/Cdk2 also triggers the activation of cyclin B/Cdk1, which leads to the G2/M transition. During mitosis, cyclin B/Cdk1 activates by phosphorylation the protein Cdc20. This protein creates a negative feedback loop involving cyclin A/Cdk2 and cyclin B/Cdk1 by promoting the degradation of these complexes. The regulations controlled by Cdc20 allow the cell to complete mitosis, and to start a new cell cycle if sufficient amounts of GF are present. The model represents a simplified version of a more detailed model proposed by Gérard and Goldbeter (2009) [31].

The dynamics of cyclin D/Cdk4-6 (*Md*), cyclin E/Cdk2 (*Me*), cyclin A/Cdk2 (*Ma*), cyclin B/Cdk1 (*Mb*), transcription factor E2F (*E2F*), and protein Cdc20 (*Cdc20*) are described by the following ordinary differential equations:

(1) $$\frac{dMd}{dt}=\mu \left(v_{sd}\cdot \frac{GF}{K_{gf}+GF}-V_{dd}\cdot \frac{Md}{K_{dd}+Md}\right)$$

(2) $$\frac{dE2F}{dt}=\mu \left(V_{1e2f}\cdot \frac{E2F_{tot}-E2F}{K_{1e2f}+E2F_{tot}-E2F}\cdot \left(Md+Me\right)-V_{2e2f}\cdot \frac{E2F}{K_{2e2f}+E2F}\cdot Ma\right)$$

(3) $$\frac{dMe}{dt}=\mu \left(V_{1Me}\cdot E2F\cdot \left(a_{1}+b_{1}\cdot Me\right)\cdot \frac{Me_{tot}-Me}{K_{1Me}+Me_{tot}-Me}-V_{2Me}\cdot Ma\cdot \frac{Me}{K_{2Me}-Me}\right)$$

(4) $$\frac{dMa}{dt}=\mu \left(V_{1Ma}\cdot E2F\cdot \frac{Ma_{tot}-Ma}{K_{1Ma}+Ma_{tot}-Ma}-V_{2Ma}\cdot Cdc20\cdot \frac{Ma}{K_{2Ma}+Ma}\right)$$

(5) $$\frac{dMb}{dt}=\begin{array}{c} \mu (V_{1Mb}\cdot Ma\cdot \left(a_{2}+b_{2}\cdot Mb\right)\cdot \frac{K_{ie}}{K_{ie}+Me}\cdot \frac{Mb_{tot}-Mb}{K_{1Mb}+Mb_{tot}-Mb}\\ -V_{2Mb}\cdot \left(a_{3}+b_{3}\cdot Wee1\right)\cdot \frac{K_{ib}}{K_{ib}+Mb}\cdot Cdc20\cdot \frac{Mb}{K_{2Mb}+Mb}) \end{array}$$

(6) $$\frac{dCdc20}{dt}=\mu \left(V_{1Cdc20}\cdot Mb\cdot \frac{\left(Cdc20_{tot}-Cdc20\right)}{K_{1Cdc20}+Cdc20_{tot}-Cdc20}-V_{2Cdc20}\cdot \frac{Cdc20}{K_{2Cdc20}+Cdc20}\right)$$

Each activation/deactivation process follows Michaelis–Menten kinetics, modulated by regulatory terms (see [33] for details). The model also accounts for the self-activation of cyclin E/Cdk2 via Cdc25 (parameter *b*_1_) and of Cyclin B/Cdk1 via Cdc25 (parameter *b*_2_) and via *Wee1* (mutual deactivation, parameters *b*_3_ and *K_ib_*). These positive feedback loops have been shown to increase the amplitude of the oscillations in the various cyclin/Cdk complexes, and to enhance the robustness of the Cdk oscillations [33]. A scaling parameter μ, which multiplies all equations, is used to adjust the autonomous period of the cell cycle.

The control by the circadian clock is incorporated by a sinusoidal function, representing the circadian oscillation in the activity of *Wee1*, explicitly added in Equation (5):

(7) $$Wee1=A\;sin\;\left(\;\frac{\pi \;t_{day}}{\tau_{clock}-\tau_{0}}\right)\;H\left(\tau_{clock}-\tau_{0}-t_{day}\right)$$

where *τ_clock_* is the period of the circadian clock (set to 24 h), *A* is amplitude (strength of circadian forcing), *τ*_0_ is the period of time during which *Wee1* is not expressed (set to 12 h, corresponding to the night phase), and *t_day_* is the time of the day (between 0 and 24 h). We refer here to the zeitgeber time, noted ZT, and ZT 0 corresponds to the beginning of the light phase. Thus ZT 0 roughly corresponds to 8 am and ZT 12 to 8 pm. *H(x)* is the Heaviside function: H takes the value 0 when *x* < 0 and the value 1 when *x* > 0. Thus, when *τ_clock_* < *τ*_0_ + *t_day_*, *Wee1* = 0, meaning that during this period of time, the level of *Wee1* is too low to exert its inhibition.

To keep the model as simple as possible, several assumptions have been made. First, the model is centered on the cyclin/Cdk complexes and their activity. We did not explicitly model the synthesis and degradation of the cyclins. We assume that, as soon as the cyclin is present, it binds its respective Cdk and once the latter is deactivated, this leads to the rapid degradation of the cyclin. Second, our model does not take into account the regulation of the basal expression of *Wee1* by the cell cycle. Third, key cell cycle regulators, such as p21/p27 or pRB/E2F, have not been included in the present model. Using a detailed version of the model, Gérard and Goldbeter (2009, 2012) previously showed that oscillations of the Cdk network only occur when the levels of the antagonistic proteins pRB and E2F are properly balanced and in the presence of a sufficient amount of GF [31,34]. Computational simulations further showed that in cancer cells, oscillations occur for a larger range of pRB/E2F concentrations and are largely independent of GFs. Here, we focus on circadian entrainment and thus opted for a simple model. A complete understanding of the behavior of cancer vs. healthy cells upon circadian control would however require a more comprehensive model including these key regulators.

The parameter values are listed in Table S1. The simulations and time series analyses were performed with Matlab (ode45 solver).

## 3. Results

### 3.1. Dynamics of the Cdk Network and Sensitivity Analysis

**Figure 2:**
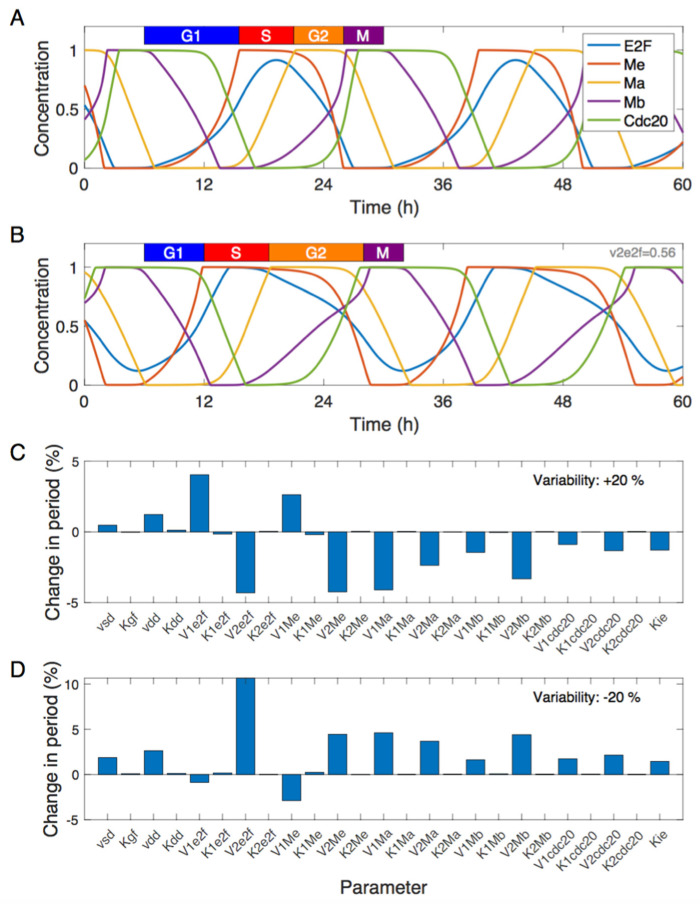
(**A**) Dynamics of the Cdk network obtained by numerical simulation of Equations (1)–(6) with the default parameter values (Table S1, μ = 0.3718). (**B**) Dynamics of the Cdk network obtained when the value of *v_2e2f_* was decreased by 20% (*v_2e2f_* = 0.56). (**C**,**D**) Sensitivity analysis showing changes in oscillation period when each parameter value was increased (**C**) or decreased (**D**) by 20%.

Figure 2A shows the dynamics of the Cdk network. In the presence of growth factor (GF = 1), Cdk/cyclin complexes are sequentially and periodically activated (see also [33]). The autonomous period of the cell cycle for the default parameter values was assumed to be, and set at, 24 h. This was done by adjusting the scaling parameter μ to μ = 0.3718 (default value). The S phase can roughly be associated with periods of high activity of cyclin E/Cdk2 (*Me ≈ 1* and *Me* > *Ma*) whereas the M phase corresponds to periods of high activity of cyclin B/Cdk1 (*Mb* ≈ 1 and *Me* > *Cdc20*). These phases are separated by G1 (Cdc20 ≈ 1) and G2 (*Ma* ≈ 1).

Changing the kinetic parameter values can change the period of the oscillations. For example, decreasing *V_2e2f_* by 20% led to a longer cell cycle, characterized by a period of 26.5 h (Figure 1B). We also noticed that the duration of the different phases of the cell cycle (i.e., the length of the plateau of the Cdk/cyclin complexes and the interval between these plateaus or their overlap) were not all affected in the same way. Whereas the duration of the G1 phase was slightly reduced, the duration of the S and G2 phases, were extended, leading to an overall longer period. The amplitude of the oscillations however, were not strongly altered. Indeed, each cyclin/Cdk variable reached its maximum around 1 for a certain period of time. This is likely due to the positive feedback loops, as discussed in [33].

A sensitivity analysis was conducted to determine the extent of the change in oscillation period when the same level of variability (+20% or −20%) was applied on each of the 25 kinetic parameters, including (in) activation rates of the cyclin/Cdk complexes and the Michaelian constants (Figure 2C,D). These results showed that the different parameters have varying degrees of influence on the oscillation period. The effect of a positive variability was generally opposite to that of a negative variability, but the extent of the effect depended on each parameter. Increasing the value of a parameter mostly led to a decrease in period, and vice versa.

### 3.2. Entrainment of the Cell Cycle by the Circadian Clock

**Figure 3:**
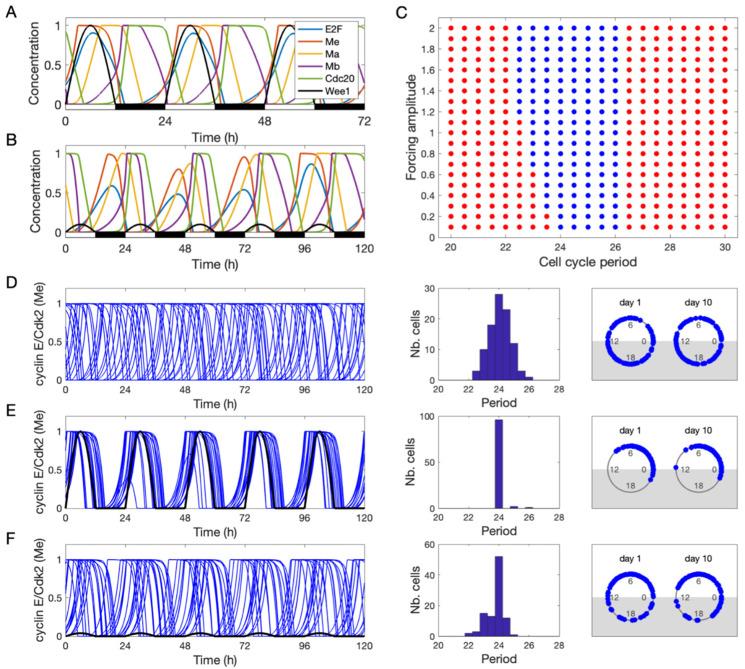
Entrainment of the cell cycle by the circadian clock. (**A**) Entrainment of the cell cycle to 24 h period when the autonomous period of the cell cycle was very close to 24 h (μ = 0.3718) and when the amplitude of the circadian input was sufficiently high (*A* = 1). (**B**) Lack of entrainment of the cell cycle when the autonomous period was set to 29.75h (μ = 0.3) and when the amplitude of the circadian input was low (*A* = 0.1). (**C**) Entrainment (blue dots) vs. non-entrainment (red dots) as a function of the autonomous cell cycle period and of the circadian amplitude. Entrainment was achieved if oscillations were phase locked and had a constant amplitude. (**D**) Time evolution of a population of cells with a variability of 10% applied on all parameters. More specifically, the value of each parameter was changed by a value of *x*% with *x* randomly selected in the range [−10, 10]. The dynamics of *Me* is shown for 15 cells taken from a population of 100 cells. (**E**) Circadian entrainment of the same population of cells (*A* = 1). (**F**) Partial entrainment of the same population of cells at a low circadian amplitude (*A* = 0.1). The black curves in panels (**E**,**F**) denote the profile of *Wee1* activity. On the right of each time series are the distributions of the periods and of the phases on day 1 and day 10. Phase 0 corresponds to the dark–light transition and the phase of each oscillator was determined by the time of day at which the maximum of variable *Me* was reached.

The impact of the circadian clock on the cell cycle can be simulated by considering a circadian forcing by *Wee1*. When *Wee1* undergoes oscillations with a large amplitude (*A* = 1) and 24 h period, the cell cycle was entrained (Figure 3A). No change was noticeable in the dynamics because the autonomous period of the cell cycle was already very close to 24 h. However, if the period of the cell cycle was arbitrarily set to 29.75 h (μ = 0.3), and if the amplitude of the circadian forcing was too low (due, for example, to perturbations in the clockwork on in the coupling mechanism), then the cell cycle was not entrained by the circadian clock (Figure 3B). This lack of entrainment was manifested by day-to-day changes in the amplitude of the oscillations and by an absence of phase locking: the maximum of a given cyclin/Cdk complex did not occur at the same time every day.

Proper entrainment (with phase locking) thus depends on the amplitude of the circadian forcing and on the autonomous period of the cell cycle. We systematically investigated the range of conditions leading to entrainment as a function of these two parameters (Figure 3C). The results of this systematic analysis confirmed that a cell cycle with a period close to 24 h was easier to entrain, regardless of the forcing amplitude. The range of the autonomous period for successful entrainment was about 23–26 h for the default circadian amplitude of 1. The required circadian amplitude depends on the autonomous period: the shorter the cell cycle, the higher the required amplitude. Cell cycles with a period longer than 26 h appeared difficult to entrain. We also observed that oscillations with autonomous periods shorter than 24 h were easier to entrain than those with periods longer than 24 h. This range of entrainment is called an Arnold tongue and was studied in more detail in [34]. The latter study showed that multiple inputs from the circadian clock to the cell cycle do not necessarily facilitate entrainment.

The cell cycle is subject to intercellular variability. One way to account for this variability is to incorporate variability into the parameter values. As discussed above, changing parameter values typically affects the period of the cell cycle. We considered a heterogeneous population of cells by applying, for each cell, some variability on all parameters. For a given cell, the value of each parameter was increased or decreased by a small percentage, randomly selected within a certain range. In absence of circadian input, the cells were rapidly desynchronized (Figure 3D). When the circadian signal was applied on the same population of cells, the large majority of cells were entrained and phase locked, i.e., they maintained their phase over several days (Figure 3E). Due to the variability, however, they did not all divide perfectly in phase. This reflects the behavior of a population of healthy cells. Cancer cells, on the contrary, are assumed to have no/low circadian input. In the absence of coupling, the cells were completely desynchronized from the circadian clock (Figure 3D). In other words, they divided at any time of the day. In the presence of a weak coupling, although the cells tended to have a period close to 24 h, they were not phase locked and not synchronized with each other (Figure 3F). A certain proportion of cells laid outside the entrainment region (Arnold tongue). Contrarily to the case of uncoupled cells, the phase distribution here was not homogeneous, and some phases were more frequent than others. In other words, cell division may still occur more frequently at certain times of the day.

Cyclin B/Cdk1 is not the only target of *Wee1*. *Wee1* also inhibits the Cyclin E/Cdk2 complex [46,47]. In Figure S1, we compare the dynamics of the cell cycle in the absence of any circadian signal (Figure S1A), with a *Wee1*-mediated circadian input only on Cyclin B/Cdk1 (Figure S1B), with a *Wee1*-mediated circadian input only on Cyclin E/Cdk2 (Figure S1C), and with a *Wee1*-mediated circadian input both on Cyclin B/Cdk1 and on Cyclin E/Cdk2 (Figure S1D). When only one entry point was considered, the oscillations were well entrained. However, we noticed that the phase of the oscillations was shifted by nearly 12 h depending on the targeted Cyclin/Cdk complex (panel C vs. panel B). When *Wee1* simultaneously inhibited both complexes, entrainment was lost. This is likely explained by the fact that each forcing tends to set a different entrainment phase. This result is in agreement with the conclusion previously reported in [34]: multiple periodic forcing does not necessarily facilitate entrainment.

Proper entrainment of the cell division cycle thus depends on the strength of circadian signal, on the kinetic parameters, and on the coupling mechanism. A lack of entrainment, as may occur in cancer cells, results in a cell division cycle that is not synchronized with the time of the day. In the next section, we examine the consequences of this impaired synchronization for chronotherapy.

### 3.3. Simulating Chronotherapeutic Treatments

The fact that cancer cells are not or less effectively synchronized by the circadian clock may be exploited to address the question of the time-dependent effectiveness of anti-cancer treatments. More specifically, a drug that targets cells during DNA replication, such as 5-fluorouracil (5-FU), should be administrated at the time where healthy cells are unlikely to be in S phase. Due to the lack of synchronization of cancer cells, we may expect a certain fraction of these cells to be in S phase at that time of the day. Applying the drug at this specific time would thus reduce its toxicity while killing a fraction of the cancer cells.

**Figure 4:**
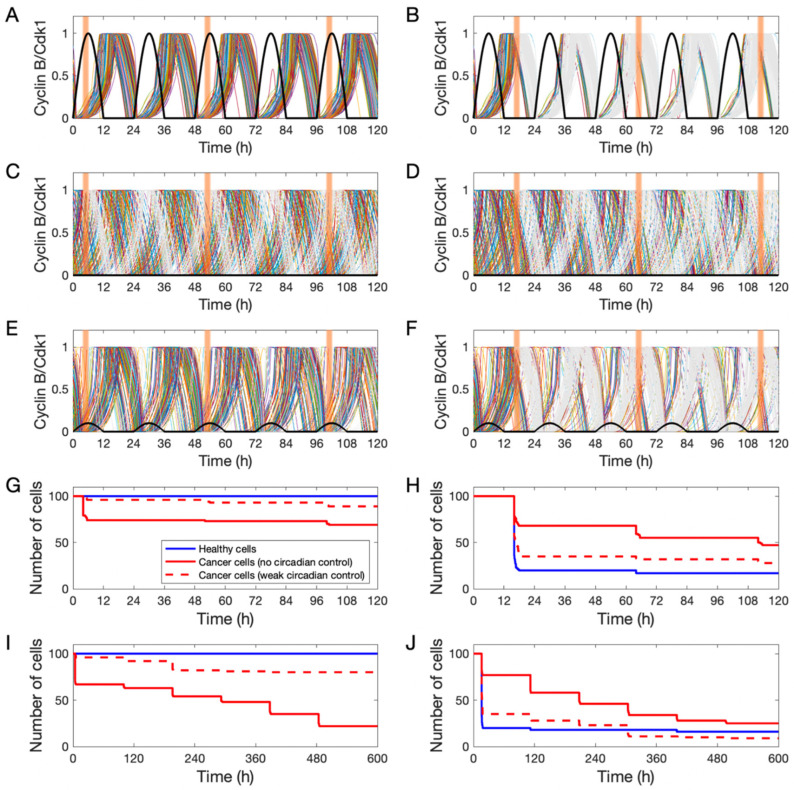
Effect of an anti-cancer drug administrated at different times. Effect of anti-mitotic drug given at ZT 4 (left panels) and at ZT 16 (right panels) on entrained (healthy) cells (panels (**A**,**B**), *A* = 1) and on non-entrained (panels (**C**,**D**), *A* = 0) or weakly entrained cells (panels (**E**,**F**), *A* = 0.1). (**G**,**J**) Evolution of the number of (living) cells over time. In these simulations, the initial number of cells was 100, the drug targets cells with a level of cyclin B/Cdk1 (*Mb*) larger than 0.95 (i.e., cells in M phase), the duration of the application of the drug was 2 h and the drug is delivered every 2 days (panels (**A**–**H**)) or every 4 days over a longer period of time (panels (**I**,**J**)). ZT 0 corresponds to the beginning of the L phase (i.e., start of expression of *Wee1*). The black curves in panels (**A**–**F**) denote the profile of *Wee1* activity. The orange bars indicate the time and duration of the application of the drug. In panels (**A**–**F**), the color curves are the levels of *Mb* in living cells, while the grey curves indicate the (virtual) profile of *Mb* in dead cells, i.e., the profile of *Mb* is the cells that have been targeted (and thereby killed) by the anti-mitotic drug. The fraction of living cells is number of cells that were not exposed to the drug while having a high level of Cyclin B/Cdk1, divided by the initial number of cells (100 in the present case).

To simulate the action of a cell cycle phase-specific drug, we assumed that a cell will be killed if it is in the target phase of that drug during the period of application of the drug, i.e., if the level of the corresponding cyclin/Cdk complex is above a certain value. This is illustrated in Figure 4 for the case of a mitosis-targeting drug, such as paclitaxel or vinorelbine. Such a drug would kill cells in M phase, i.e., with a high level of cyclin B/Cdk1 (variable *Mb*). Depending on the time of the treatment, a different fraction of the cells will be killed. In panel A, nearly all the cells were entrained and, even if some variability in the phase of *Mb* was observed, no cell appears to enter into mitosis during the light phase (i.e., when *Wee1* is highly expressed). Thus, when the anti-mitotic drug was applied at ZT 4, no cell was killed (panel B, see also blue curve in panels G and I). On the contrary, a large fraction of cells presented a maximal activity of *Mb* at ZT 16, and, consequently, a drug administrated at ZT 16 will kill a large number of cells (see also blue curve in panels H and J). Note that since most cells were phase locked, nearly no additional cells will be killed by subsequent administrations of the drug.

Cancer cells which were not entrained by the circadian clock (*A* = 0) displayed any phase and this phase can change day after day (panel C). At any time, a certain fraction of cells will have a high level of *Mb*. Thus, regardless of the time of the treatment, a certain fraction of the cells were killed by the drug (see panels C and D and solid red curve in panels G-J). After a first exposure to the drug, about 25% of cells were killed. Subsequent treatments further killed more cells. This is clearly visible in panels I and J where the application of the drug was repeated every 4 days over a longer period of time. This is due to the fact that cells were not phase locked. A cell may thus have its maximum level of *Mb* during the night in a given day and during the light phase some days later. It would thus escape the drug during the first exposure to the drug but may be killed during a subsequent treatment. This suggests that a repeated drug application at the right time of the day with some interval between the medications (to provide time for the cancer cells to desynchronize) would be an effective strategy.

We also simulated the effect of a weak coupling to the circadian clock (panels E and F). As discussed in the previous section, upon weak circadian control, oscillations may not be efficiently entrained, but may still show some “preference” for some phases. In that case, a time dependence of the drug efficiency was still observed (see panels G-J, dashed red curves), and chronomodulated therapy may be of limited value: increasing efficiency (panels H and J, strong decays of cancer cells, dashed red curves) was coupled with a higher toxicity (quick and strong decay of healthy cells, blue curves).

**Figure 5:**
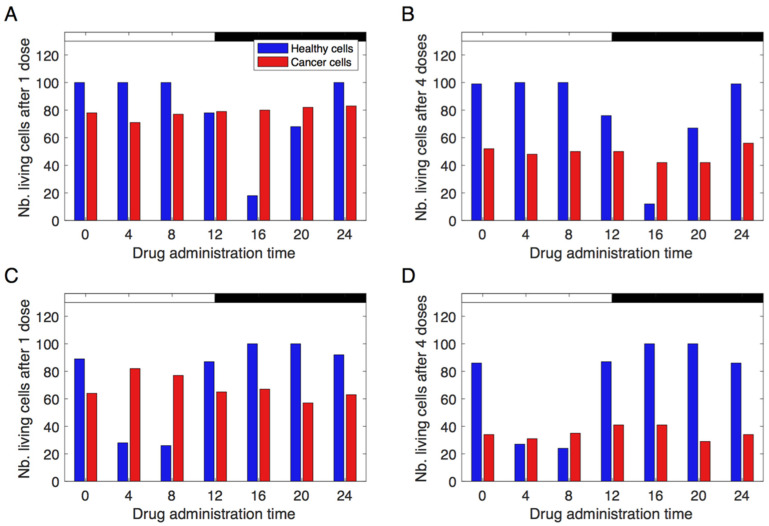
Effect of the schedule of the treatment. The different histograms give the number of living entrained/healthy (blue) vs. non-entrained/cancer cells (red) remaining after the application of an anti-*Mb* drug (panels (**A**,**B**)) or anti-*Me* drug (panels (**C**,**D**)), when a single dose (panels (**A**,**C**)) or 4 doses at an interval of 5 days (panels (**B**,**D**)) is administered. As in Figure 4, the initial number of cells is 100, the drug targets cells with a level of *Mb/Me* larger than 0.95, and the duration of the application of the drug is 0.5 h. ZT 0 represents the beginning of the L phase (i.e., start of expression of *Wee1*).

Figure 5 shows the results of a systematic analysis of the effect of a drug as a function of the administration time on properly entrained cells (healthy cells, blue bars) and on un-entrained cells (cancer cells, red bars). Figure 5A,B confirmed the results presented above (Figure 4): an anti-mitotic drug is predicted to have a high toxicity when administrated at ZT 16, with a majority of healthy cells killed after application of a single dose of the drug (panel A). For these cells, a repeated application of the drug did not significantly change the outcome (panel B). In contrast, a certain fraction of cancer cells was killed, regardless of the time of day at which the drug was given and repeating the treatments led to an increased efficacy. The model predicts that the best schedule to take such a drug is during the day phase, around ZT 0-8, in order to minimize its toxicity.

Figure 5C,D show similar results when considering a drug that targets cells in S phase (such as seliciclib or 5FU). Here, the targeted cells were the ones with a high level of *Me* when exposed to the drug. Consequently, the best schedule to give the drug was during the night phase, around ZT 16-20. Regardless of the type of drug, increasing the duration of action of the drug led to a higher number of cells killed, but the time of high toxicity remained the same (not shown).

As discussed in the previous section, *Wee1* does not only act at the level of cyclin B/Cdk1, but also inhibits the cyclin E/Cdk2 complex. To check if this alternative mode of coupling or the combination of both alter the conclusions, we computed the survival rate as a function of the administration time for the different scenarios (Figure S2). The results shown in Figure S2A,B, generated for the cases without a circadian signal (panels A) and with *Wee1* acting on cyclin B/Cdk1 (panels B), are similar to the ones shown in Figure 5A,B and serve as a reference. In Figure S2C, *Wee1* acts only on cyclin E/Cdk2. Two differences with the previous case can be highlighted. First, at nearly any time of the day, a certain percentage of the cells were killed by the drug. This is due to the fact that, in presence of heterogeneity in the kinetic parameters, a certain fraction of the cells were not entrained and exposed to the drug at any time of the day. Second, the time of higher toxicity was around ZT 4, i.e., 12 h before the time of higher toxicity found when *Wee1* was acting only on cyclin B/Cdk1. This is a consequence of the opposite phase of entrained cells (see Figure S1B vs. Figure S1C). Finally, when considering the action of *Wee1* on both cyclin B/Cdk1 and cyclin E/Cdk2, a profile similar to the one obtained with *Wee1* acting only on cyclin B/Cdk1 was found, suggesting that this entrainment mode is more efficient and dominates the dynamics. However, the range of time of high toxicity appeared longer than when *Wee1* acted only on cyclin B/Cdk1.

## 4. Discussion

Successful application of chronopharmacological treatments requires a good understanding of the behavior of the cell cycle in healthy and cancer cell populations in response to the circadian clock. Here, we used computational modelling to study the circadian forcing of the mammalian cell cycle at the population level in presence of variability in kinetic parameters. Numerical simulations allow us to determine conditions for successful entrainment and synchronization, and to highlight some aspects to take into consideration in order to predict optimal protocols for chronotherapeutic treatments.

The model was initially parameterized to generate oscillations characterized by a sequential activation of the different cyclin/Cdk complexes and with a period close to 24 h [33]. Running the model with different parameter values typically leads to changes in oscillation dynamics (period, amplitude, and, possibly, loss of oscillations). Changes in oscillation period reflect the variability in cell cycle rates under different conditions or in different cell types. A decrease in oscillation amplitude could be a cause of insufficient signaling intensity of the cyclin/Cdk network. When oscillation amplitude is low, concentrations of cyclin/Cdk complexes could not reach the threshold for signaling the next phase. Without a functioning cyclin/Cdk network, the cell cycle could no longer proceed. Disruption or loss of oscillations may have dramatic consequences such as incorrect order of progression of phases or skipping of phases, leading to unsuccessful cell replication.

A sensitivity analysis was performed to assess the influence for each parameter on the period of the oscillations. Some reactions of the cyclin/Cdk network may be more critical in governing network dynamics. The parameters *V*_1e2f_ and *V*_2e2f_ determine E2F concentration, which signals the activation of both cyclin E/Cdk2 and cyclin A/Cdk2 complexes. Not surprisingly, increasing or decreasing these kinetic rates impacted the duration of the cell cycle, but interestingly, not all the phases of the cell cycle were affected in the same way. In general, positive variability mostly led to a decrease in the period. This finding was unexpected as we anticipated that an increase in activation constants would speed up the cell cycle and decrease the period, while an increase in inactivation constants would produce the opposite result. A possible explanation for this is that each component of the cyclin/Cdk complex network is interconnected and the effect of variability in one constant will be compensated by other components.

Besides unavoidable variability on parameter values, other factors affect the duration of the cell cycle phases or, more generally, the decision to divide. The decision to enter the cell cycle from a quiescence state is a regulated process which depends on DNA damage and mitogen signals [48,49]. Here, we implicitly considered that all cells have passed the restriction point and remain in the proliferation state. In a future extension of the model, a bistable switch involving p21 and DNA damage, as described in [48], may be incorporated in our model to explicitly distinguish quiescent and proliferating cell populations.

Upon circadian forcing by *Wee1*, we were able to entrain the cell cycle to 24 h, as well as to synchronize a cell population, even in presence of some variability. Such intercellular variability, if not too high, did not prevent entrainment but impacted the entrainment phase: not all cells entered the S (or M) phase at exactly the same time, but the phases were nevertheless restricted to a limited time window. Our simulations also showed that cells with an autonomous period longer than 26 h were hard to entrain, regardless of the coupling strength, suggesting that the circadian forcing by *Wee1* was more capable of speeding up the cell cycle than slowing it down. Other modes of circadian forcing may perform better in slowing down the cell cycle. An exhaustive computational study of the coupling between the cell cycle and the circadian clock, taking into account multiple links between the two oscillators (via *Wee1*, p21, and cyclin E), revealed additional entrainment patterns and showed that multiple coupling mechanisms did not necessarily increase the range of entrainment [34]. This is in agreement with our finding that considered that the inhibition of cyclin E/Cdk2 by *Wee1* did facilitate entrainment and, consequently, led to a larger time window of high toxicity. In a future extension of the work, it will be worthwhile to include the multiple control points of the cell cycle, and to evaluate their relative importance in the entrainment of the cell cycle and in determining the entrainment phase. This is a prerequisite to developing optimal chronotherapy strategies. Related theoretical studies highlight the role of factors, such as growth factors or dexamethasone, on the entrainment pattern [35]. The possible influence of the cell cycle on the circadian clock was addressed by Yan and Goldbeter (2019) who reported an increased robustness and a reduction of complex oscillations when considering a bidirectional coupling [50].

Cancer cells may be fully decoupled from the circadian clock or may have a disrupted clock. In both cases, the cell cycle runs autonomously. Due to the cellular variability, at the level of a population, the cells were rapidly desynchronized and divided at any time of the day. To mimic a weak circadian control, we also simulated a cancer cell population by lowering the circadian amplitude. Although the cell population was less synchronized than healthy cells, most of the resulting cell cycle periods remained close to 24 h. These cells divided at any time of the day but, in contrast to the case of complete decoupling, the cells had a higher propensity to enter S (or M) phase at specific times of the day.

Simulations of the administration of anti-cancer drugs to healthy cell populations allowed us to assess drug effectiveness and toxicity as a function of the administration time. Due to their proper entrainment and good synchronization, healthy cells showed periods of high sensitivity and periods of insensitivity to drugs. On this basis, the model allows us to predict the administration time to be avoided to minimize toxicity. If cancer cells are fully decoupled from the circadian clock, no optimal timeframe which maximizes effectiveness could be identified because cell divisions are randomly and homogeneously distributed over the day. In this case, a repeated treatment may be advocated because cells that escape the drug one day may be exposed to the drug some days later. The interval between the treatments should be sufficiently long to allow cancer cells running with their own period to get desynchronized with respect to the time of the day. However, when cancer cells are weakly coupled to the circadian clock, they may still divide preferentially at some times of the day. This allows us to find a time of maximum efficiency, but this time may unfortunately coincide with the time of maximum toxicity.

Other features that should be taken into account in further developments of the model are the pharmacokinetics/pharmacodynamics (PK/PD) characteristics of the drug. Here, we arbitrarily applied treatments for 2 h and we assumed instantaneous effect on cells in the target phase. For future applications, it will be necessary to determine the half-life of the drug of interest, as well as its absorption and transport kinetics and possibly its molecular interaction with cell cycle components or with signaling pathways. Taking into account the lag time between the administration of the drug and its effect in the targeted organ will induce a time shift (advance) in the optimal administration profile. Similarly, the duration of activity of the drug, which may be organ-specific and may be dependent on the dose, should also be quantified. It will be also crucial to determine precisely the phase during which a given cell is sensitive to the drug and the level of lethality of the drug. Once all these data are available, the current model may be adapted or extended to incorporate a PK/PD module to model drug action in order to make quantitative predictions.

## 5. Conclusions

Computational modelling is a powerful approach to explore the dynamics of complex processes like the cell cycle and its entrainment by the circadian clock, and can be used to predict optimal chronotherapeutic protocols [38,39,40,41,42,51,52]. These studies rely on pharmacokinetics/pharmacodynamics data and on fitting of concentration time profiles to estimate the values of kinetic parameters. Calibrated molecular models are then used to identify key regulators of the cell cycle–circadian clock dynamics or to design optimal protocols for drug administration. Inter-individual and organ-specific differences, as well as stochastic variability is taken into account in fitting procedures but the inter-cellular variability resulting in heterogeneous cell populations is not considered. In line with other multiscale approaches [53,54], the present work highlights the need to better characterize inter-cellular variability in the dynamics of the cell cycle and its consequence for circadian entrainment. Fully calibrated multi-scale models integrating PK/PD aspects and population-level dynamics will then have a great potential to design—and possibly personalized—cancer treatments.

## Data Availability

Not applicable.

## Supplementary Materials

The following supporting information can be downloaded at: https://www.mdpi.com/article/10.3390/biology12040612/s1, Table S1. Parameters values; Figure S1. Dynamics of the cell cycle network; Figure S2. Effect of the schedule of the treatment.
