# Supplementary material for: Modeling the Circadian Control of the Cell Cycle and Its Consequences for Cancer Chronotherapy
Source: Biology (Basel). 2023 Apr 18;12(4):612. doi: 10.3390/biology12040612 (PMC10135823; doi:10.3390/biology12040612)
Supplement: Supplementary file 1 [file biology-12-00612-s001.zip › biology-2294222-supplementary.pdf]

biology MDPI

Supplementary information

Modeling the circadian control of the cell cycle and its consequences for cancer chronotherapy

Courtney Leung, Claude Gérard and Didier Gonze

Table S1. Parameters values.

Symbol	Definition	Value
$Cdc20_{tot}$	Total concentration of protein Cdc20	1 μM
$E2F_{tot}$	Total concentration of transcription factor E2F	1 μM
GF	Growth factor	1 μM
Me_{tot}	Total concentration of cyclin E/Cdk2	1 μM
Ma_{tot}	Total concentration of cyclin A/Cdk1	1 μM
Mb_{tot}	Total concentration of cyclin B/Cdk1	1 μM
K_{ib}	Inhibition constant for Wee1 inactivation by cyclin B/Cdk1	0.5 μM
v_{sd}	Maximum rate of synthesis of cyclin D/Cdk4-6 induced by growth factor	0.175 $\mu M \cdot h^{-1}$
K_{gf}	Michaelis constant for synthesis of cyclin D/Cdk4-6 complex induced by growth factor	0.1 μM
V_{dd}	Maximum degradation rate of cyclin D/Cdk4-6 complex	0.245 $\mu M \cdot h^{-1}$
K_{dd}	Michaelis constant for degradation of cyclin D/Cdk4-6 complex	0.1 μM
V_{1e2f}	Rate constant for activation of E2F by cyclin D/Cdk4-6 and cyclin E/Cdk2 complexes	0.4 h^{-1}
K_{1e2f}	Michaelis constant for E2F activation by cyclin D/Cdk4-6 and cyclin E/Cdk2 complexes	0.005 μM
V_{2e2f}	Rate constant for inactivation of E2F by cyclin A/Cdk2 complex	0.7 h^{-1}
K_{2e2f}	Michaelis constant for E2F inactivation by cyclin A/Cdk2 complex	0.005 μM
V_{1Me}	Rate for activation of cyclin E/Cdk2 by E2F	1.1 $\mu M \cdot h^{-1}$
K_{1Me}	Michaelis constant for cyclin E/Cdk2 activation by E2F	0.005 μM
V_{2Me}	Rate constant for inactivation of cyclin E/Cdk2 by cyclin A/Cdk2	1.6 h^{-1}
K_{2Me}	Michaelis constant for cyclin E/Cdk2 inactivation by cyclin A/Cdk2	0.005 μM
V_{1Ma}	Rate constant for activation of cyclin A/Cdk2 by E2F	0.6 h^{-1}
K_{1Ma}	Michaelis constant for cyclin A/Cdk2 activation by E2F	0.005 μM
V_{2Ma}	Rate constant for inactivation of cyclin A/Cdk2 by Cdc20	0.6 h^{-1}
K_{2Ma}	Michaelis constant for cyclin A/Cdk2 inactivation by Cdc20	0.005 μM
V_{1Mb}	Rate for activation of cyclin B/Cdk1 by cyclin A/Cdk2	1 $\mu M \cdot h^{-1}$
K_{1Mb}	Michaelis constant for cyclin B/Cdk1 activation by cyclin A/Cdk2	0.005 μM
V_{2Mb}	Rate for inactivation of cyclin B/Cdk1 by Cdc20	0.8 h^{-1}
K_{2Mb}	Michaelis constant for cyclin B/Cdk1 inactivation by Cdc20	0.005 μM
V_{1Cdc20}	Rate constant for activation of Cdc20 through phosphorylation by cyclin B/Cdk1	1.8 h^{-1}
K_{1Cdc20}	Michaelis constant for Cdc20 activation through phosphorylation by cyclin B/Cdk1	0.005 μM
V_{2Cdc20}	Rate of inactivation of Cdc20 through dephosphorylation	0.6 $\mu M \cdot h^{-1}$
K_{2Cdc20}	Michaelis constant for Cdc20 inactivation through dephosphorylation	0.005 μM
K_{ie}	Michaelis constant for cyclin B/Cdk1 inactivation by cyclin E/Cdk2	0.2 μM
a_1	Basal term for activation of cyclin E/Cdk2	1
a_2	Basal term for activation of cyclin B/Cdk1	1
a_3	Basal term for inactivation of cyclin B/Cdk1	1
b_1	Term representing self-activation of cyclin E/Cdk2 through mutual activation between cyclin E/Cdk2 and phosphatase Cdc25	1
b_2	Term representing self-activation of cyclin B/Cdk1 through mutual activation between cyclin B/Cdk1 and phosphatase Cdc25	1
b_3	Term representing deactivation of cyclin B/Cdk1 enhanced by Wee1	20

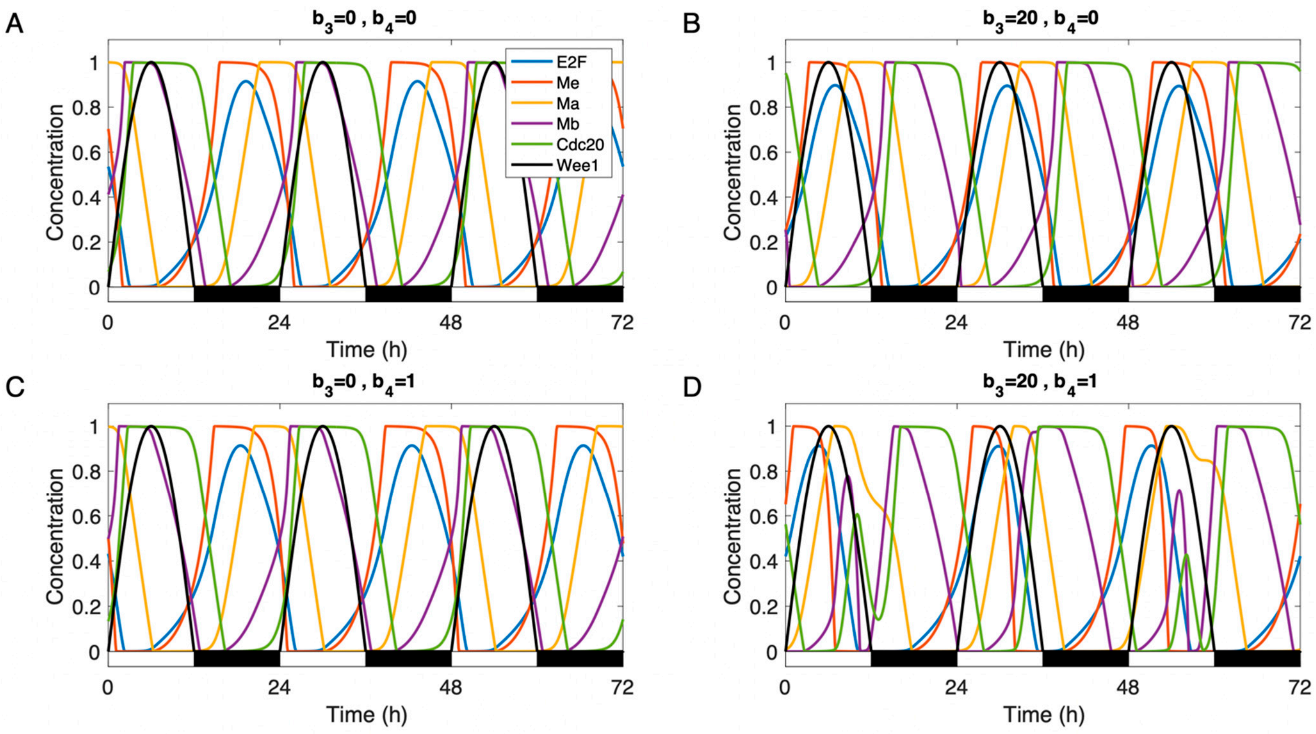

Figure S1. Dynamics of the cell cycle network (A) in absence of circadian signal, and (B-D) in presence of a circadian signal (Wee1). Three coupling scenarios are considered: Wee1 acting only on cyclin B/Cdk1 (B), only on cyclin E/Cdk2 (C), or both on cyclin B/Cdk1 and on cyclin E/Cdk2 (D). To account for the effect of Wee1 on Cyclin E/Cdk2, the term V_{2Me} in Eq. (3) has been replaced by $V_{2Me}\ (a_4 + b_4\ .Wee1)$ with $a_4 = 1$. The values of b_3 and b_4, which quantify the strength of circadian coupling, are indicated on the top of each panel. Note that the results are not qualitatively changed if we would take $b_4 = 20$ (not shown).

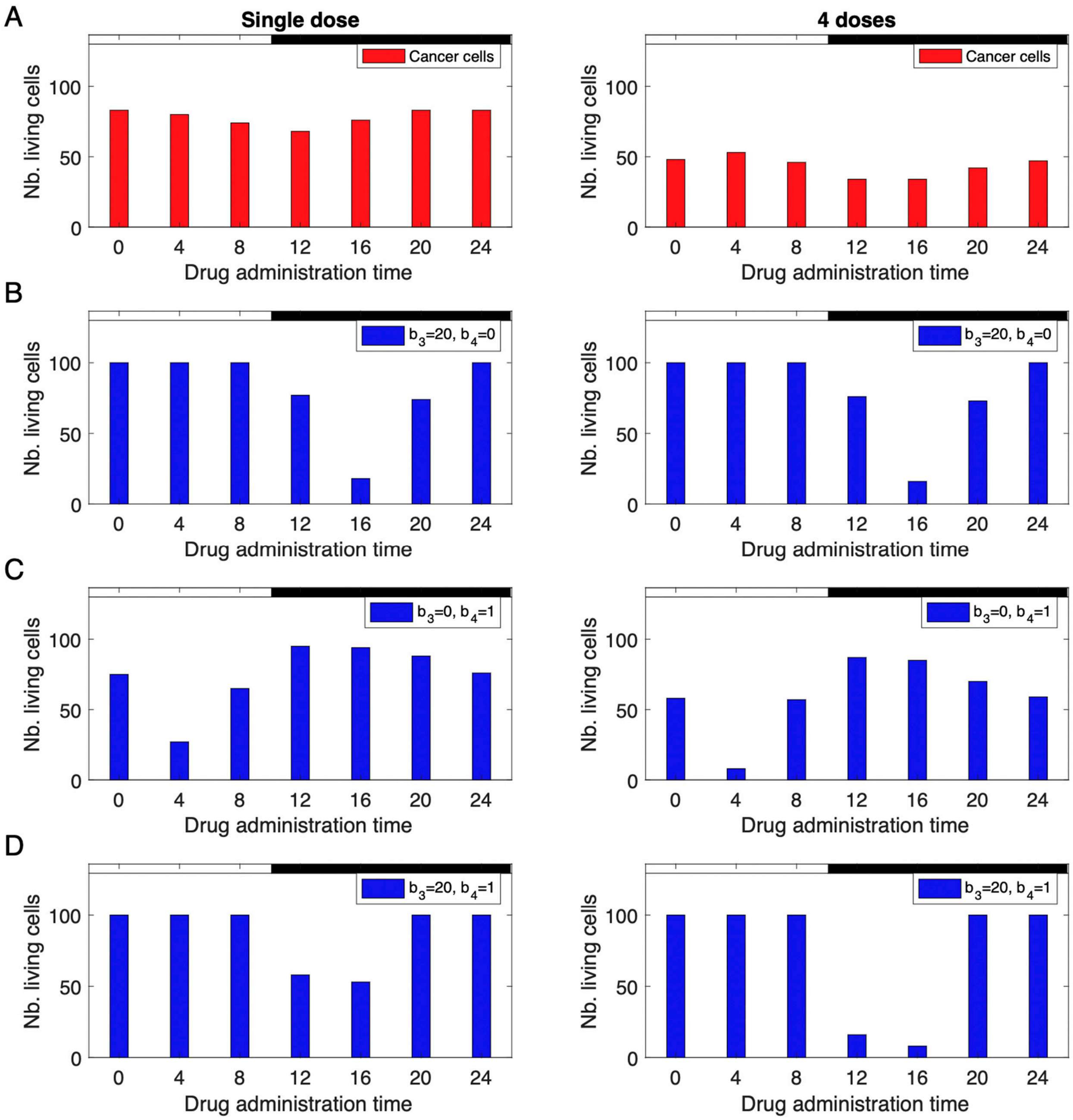

Figure S2. Effect of the schedule of the treatment. The different histograms give the number of living entrained/healthy (blue) vs non-entrained/cancer cells (red) remaining after the application of an anti-*Mb* drug, when a single dose (left panels) or 4 doses at interval of 5 days (right panels) are administrated. The following cases are considered: (A) no entrainment. (B) Wee1 acts only on cyclin B/Cdk1. (C) Wee1 acts only on cyclin E/Cdk2. (D) Wee1 acts both on cyclin B/Cdk1 and on cyclin E/Cdk2. To account for the inhibition of cyclin E / Cdk2 by Wee1, Eq. (3) has been modified as described in the legend of Figure S1. The initial number of cells is 100. The drug targets cells with a level of *Mb* larger than 0.95 and the duration of the application of the drug is 0.5h. ZT 0 represents the beginning of the L phase (i.e. start of expression of Wee1).
